# Moving fluid biomarkers for Alzheimer’s disease from research tools to routine clinical diagnostics

**DOI:** 10.1186/s13024-021-00430-x

**Published:** 2021-02-19

**Authors:** Henrik Zetterberg, Kaj Blennow

**Affiliations:** 1 Department of Psychiatry and Neurochemistry, Institute of Neuroscience & Physiology, Sahlgrenska Academy, University of Gothenburg, Mölndal, Sweden; 2 Clinical Neurochemistry Laboratory, Sahlgrenska University Hospital, Mölndal, Sweden; 3 Department of Neurodegenerative Disease, UCL Institute of Neurology, Queen Square, London, UK; 4 UK Dementia Research Institute at UCL, London, UK

**Keywords:** CSF, Plasma, Biomarkers, Alzheimer’s disease, Research, Clinical diagnostics

## Abstract

Four fluid-based biomarkers have been developed into diagnostic tests for Alzheimer’s disease (AD) pathology: the ratio of 42 to 40 amino acid-long amyloid β, a marker of plaque pathology; total-tau and phosphorylated tau, markers of AD-related changes in tau metabolism and secretion; and neurofilament light, a marker of neurodegeneration. When measured in cerebrospinal fluid, these biomarkers can be used in clinical practice to support a diagnosis of mild cognitive impairment or dementia due to AD. Recently, technological breakthroughs have made it possible to measure them in standard blood samples as well. Here, we give an updated account of the current state of the fluid-based AD biomarker research field. We discuss how the new blood tests may be used in research and clinical practice, and what role they may play in relation to more established diagnostic tests, such as CSF biomarkers and amyloid and tau positron emission tomography, to facilitate the effective implementation of future disease-modifying therapies.

## Background

Alzheimer’s disease (AD) is a slowly progressive neurodegenerative disease that currently lacks effective treatment. The first detectable pathology of the disease is the accumulation of 42 amino acid-long amyloid β (Aβ) protein in extracellular plaques in the brain, which occurs decades before clinical symptom onset [1]. Biomarker studies suggest that Aβ accumulation is followed by increased phosphorylation and secretion of tau [2], a microtubule-binding axonal protein that is highly expressed in cortical neurons [3]. This dysfunctional tau metabolism is strongly associated with neuronal degeneration with the development of intraneuronal neurofibrillary tangles that are composed of hyperphosphorylated and truncated tau proteins [4]. Neurodegeneration eventually translates into the AD clinical syndrome, with cognitive symptoms that worsen as the disease progresses [5]. Four fluid-based biomarkers have been developed into diagnostic tests for these essential brain changes in the AD process: the ratio of 42 to 40 amino acid-long amyloid β peptides (Aβ42/Aβ40), a marker of plaque pathology; total-tau and phosphorylated tau (T-tau and P-tau, respectively), markers of AD-related changes in tau metabolism, phosphorylation and secretion; and neurofilament light (NfL), a marker of neurodegeneration [6]. Originally, these biomarkers could only be measured in cerebrospinal fluid (CSF), but technological progress resulting in improved analytical sensitivity has now made it possible to measure them in standard blood samples as well.

**Fig. 1:**
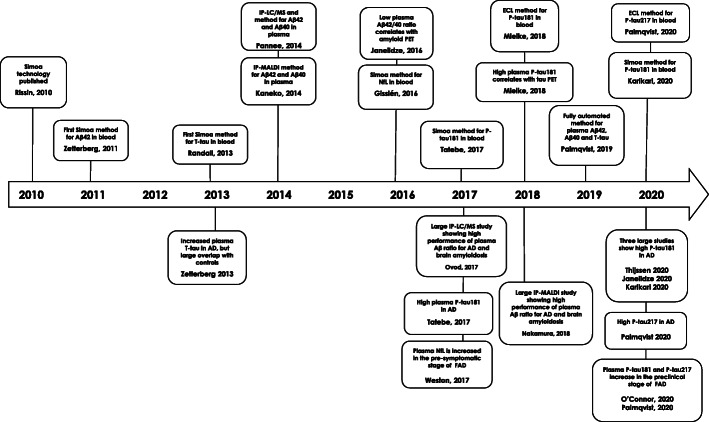
Timeline for blood biomarker developments during the last decade. Abbreviations: Simoa, Single molecule array; Aβ42, the 42 amino acid form of amyloid β; T-tau, total-tau; IP-LC/MS, immunoprecipitation liquid chromatography-mass spectrometry; IP-MALDI, immunoprecipitation matrix-assisted laser desorption/ionization; Aβ40, the 40 amino acid form of amyloid β; NfL, neurofilament light; P-tau181, tau phosphorylated at amino acid 181; P-tau217, tau phosphorylated at amino acid 217; ECL, electrochemiluminescence

Here, we provide an overview of the biomarkers that reflect the core components of AD pathology, including biomarkers for Aβ and tau pathology and neurodegeneration, in line with the amyloid (A), tau (T) and neurodegeneration (N) classification scheme for AD biomarkers [7]. We describe the work that led to clinical implementation of the CSF biomarkers. We also provide a timeline for recent biomarker developments aimed at developing clinically implementable and easy-to-use blood tests for AD (Fig. 1). Finally, the most important biomarker measurement technologies for the AD biomarkers are summarized in Table 1.

**Table 1:** General description of the most important measurement technologies for Alzheimer’s disease biofluid-based biomarkers

Technology	Explanation
Sandwich enzyme-linked immunosorbent assay (ELISA)	The target analyte is captured between two antibodies (capture and detection). The capture antibody is immobilized onto a surface (often the plastic surface of a well, e.g., in a 96-well plate). The detection antibody is labeled with an enzyme that produces a measurable signal (fluorescence or color) by converting a substrate to a product. The lower limit of quantification of an ELISA depends on the antibodies and the target analyte but is often in the nano- to picomolar range.
Immunoassay with electrochemiluminescence detection (ECL)	A variant of ELISA but instead of an enzyme, the detection antibody is labeled with a molecule that directly produces luminescence during an electrochemical reaction. This detection principle is often a little bit more sensitive than ELISA.
Single molecule array (Simoa)	This is a classical sandwich ELISA, but the capture antibody is conjugated to magnetic beads instead of the bottom of a 96-well plate, and the sandwich complexes (bead, capture antibody, target analyte and enzyme-labeled detection antibody) are pulled down in microwells (one bead per well), where the detection reaction is allowed to occur. This compartmentalized detection reaction in a very small volume allows for the detection of the biomarker at the single molecule level. In biofluids, the Simoa assays can be 100 to 1000 times as sensitive as a regular ELISA (subfemtomolar analytical sensitivity).
Immunoprecipitation mass spectrometry (IP-MS)	This technology has been particularly useful for the development of reliable plasma amyloid β tests. Antibodies against the target analyte are conjugated to beads, and the target analyte is isolated from the sample by immunoprecipitation, eluted, and then quantified by mass spectrometry, together with an isotope-labeled internal standard. The mass spectrometric detection makes the assay very specific for the target analyte.

## Fluid biomarkers for Aβ pathology

Extracellular deposition of Aβ into plaques is the key pathological feature of AD, and has been proposed as a major pathogenic event in the disease [8]. The development of tools to measure Aβ pathology *in vivo* and prior to autopsy via biomarkers in CSF started in the 1990s [9], but it was not until 2020 that full standardization of CSF Aβ42 measurement was achieved, through the use of certified reference materials and methods [10].

AD CSF is characterized by a 50 % reduction in the concentration of the 42 amino acid-long and aggregation-prone form of Aβ (Aβ42) [11]. Aβ42 is a secreted cleavage product of amyloid precursor protein (APP) that normally is mobilized from the brain interstitial fluid into the CSF and blood, likely via the glymphatic system [12]. In AD, Aβ42 aggregates in the brain parenchyma, resulting in reduced CSF levels of the protein [13]. The diagnostic accuracy for Aβ pathology can be increased by dividing the concentration of aggregation-prone Aβ42 by the concentration of soluble Aβ40 as a normalizer for inter-individual differences in Aβ production [14]. The CSF Aβ42/Aβ40 ratio is close to 100 % concordant with amyloid positron emission tomography (PET), irrespective of which PET ligand that is used [14], and discordant subjects, who are typically CSF-positive and PET-negative, often turn PET-positive within a few years [14–16].

For many years, there was not much hope for a reliable blood test for cerebral Aβ pathology [11], but recent findings suggest that plasma Aβ42 in ratio with Aβ40 (measured by immunoprecipitation mass spectrometry or ultrasensitive enzyme-linked immunosorbent assays) reflects cerebral Aβ pathology with relatively high accuracy against both amyloid PET and CSF Aβ42/Aβ40 ratio [17–20]. A recent validation study utilizing a fully automated immunoassay (Elecsys) to measure plasma Aβ42 and Aβ40 further underscores the promising capability of plasma Aβ in clinical laboratory practice [21]. Easy-to-use protocols for pre-analytical sample handling, compatible across all plasma biomarkers for AD, have also been published [22].

While the technological developments described above have been important for showing high diagnostic performance of plasma Aβ to identify AD and brain amyloidosis, a contributing factor is likely that most new studies have used amyloid PET as the reference standard. This reduces the risk of evaluating the diagnostic performance on amyloid-negative “probable AD” patients versus cognitively unimpaired elderly who may have pre-symptomatic Aβ pathology. Having a proportion of misdiagnosed cases and controls will markedly reduce the chance to find differences in plasma Aβ, given that the Aβ42/Aβ40 ratio is reduced by only 14–20 % in plasma [17–20], compared with 50 % in CSF [11]. A complicating factor for plasma Aβ tests is also that the correlation between plasma and CSF levels is weak, which could be explained by production of Aβ peptides in platelets and other non-cerebral tissues. Nevertheless, the concordant research findings using high-precision analytical tools still represent an important research advancement towards clinical implementation, perhaps using staged testing (e.g., an Aβ test in blood favoring sensitivity over specificity, followed by a more specific CSF- or imaging-based test in memory clinics).

## Biomarkers for tau pathology

The aggregation of hyperphosphorylated forms of the axonal protein tau in the neuronal soma, forming neurofibrillary tangles, is a key pathological feature of AD, although tau inclusions in neurons or glial cells are also found in some non-AD neurodegenerative dementias, e.g., progressive supranuclear palsy and some forms of frontotemporal dementia [23]. Together with the Aβ42/Aβ40 ratio, CSF T-tau and P-tau have been proposed as biomarkers to biologically define AD [24], and are considered diagnostic in the research criteria for AD [25]. Both CSF T-tau and P-tau concentrations reflect AD-related pathophysiology, but do not reflect tau pathology in non-AD tauopathies [26, 27]. The most likely explanation for this is that the increased CSF levels of tau are due to increased phosphorylation and secretion of tau from neurons, as a neuronal response to Aβ exposure [28, 29]. In the non-AD tauopathies, tau aggregation appears to occur in neurons, and sometimes other cell types as well, without this being reflected in the extracellular fluid, at least not when the currently available T-tau and P-tau assays are used. CSF T-tau and P-tau may thus be regarded predictive markers of AD-type neurodegeneration and tangle formation, but not direct markers of these processes (and not markers of non-AD tauopathies, for which improved biomarkers are needed). However, CSF T-tau also increases in disorders with rapid neurodegeneration without amyloid or tau pathology, specifically in Creutzfeldt-Jakob disease [30] and in acute conditions such as stroke and brain trauma [31, 32], suggesting that it also may reflect neuronal injury in these conditions. Fully automated T-tau and P-tau assays for clinical use are available [33, 34], and standardization work is ongoing in collaborative efforts between the International Federation of Clinical Chemistry and Laboratory Medicine (IFCC) and the Global Biomarker Standardization Consortium (GBSC).

Regarding which phospho-form of tau to measure, the three main competitors are P-tau181, P-tau217 and P-tau231. P-tau181 is the classical AD biomarker, whilst P-tau231 was suggested to improve the differentiation of AD from frontotemporal dementia [35]; they now seem to perform similarly well in this regard. Recent data suggest that CSF P-tau217 may correlate more strongly with tau pathology determined by PET and increase earlier in response to Aβ pathology than CSF P-tau181 [36], intriguing observations that warrant additional research.

Whilst ultrasensitive plasma T-tau assays can detect neuronal injury in acute brain disorders, such as stroke and traumatic brain injury [37, 38], similarly to when T-tau is measured in CSF (see above), they work relatively poorly in AD settings [39], and the correlation with CSF is weak [40]. A potential explanation for this is that the assay set up may be vulnerable to proteolytic degradation of tau in the blood (the half-life of tau measured using currently available T-tau assays is 10 hours [41], compared with around 20 days in CSF [29]; detection of small tau fragments resistant to further proteolytic degradation may be a viable way forward towards a reliable tau assay in blood). Another possibility is that currently available T-tau assays in blood may measure peripheral tau; measuring a phospho-form of tau might make the test more CNS-specific.

Recently, we have seen a number of real breakthroughs in the plasma tau biomarker field. In 2017, Tatebe et al.. reported on the quantification of P-tau181 concentration in AD plasma with increased levels compared with control samples for which most had concentrations below the lower limit of quantification, and a good correlation between plasma and CSF P-tau levels [42]. Mielke et al.. used an immunoassay with electrochemiluminescence (ECL) detection and demonstrated a correlation between P-tau181, and amyloid and tau PET, which indicates that plasma P-tau181 is a good biomarker for brain AD pathology [43]. Using the same ECL immunoassay, these findings were replicated in a study by Palmqvist et al.., demonstrating that plasma P-tau181 associates with amyloid PET positivity and correlates strongly with CSF P-tau181 [2]. Interestingly, the change in plasma P-tau181 became significant before amyloid PET, but after CSF and plasma Aβ42, i.e., already at sub-PET threshold Aβ pathology [2]. Thus, plasma P-tau181 might be useful both diagnostically to detect early Aβ-related tau dysmetabolism, as well as for disease staging. Recent large validation studies show very similar results [44–47], corroborating plasma P-tau as a robust blood biomarker for AD pathology that should be relatively easy to standardize and implement in clinical laboratory practice.

Most data currently available suggest that P-tau217 is earlier and more strongly associated with AD pathology than plasma P-tau181 [47, 48], but more head-to-head comparisons are needed before a conclusion can be reached. As an example, while plasma P-tau217 measured by the ECL immunoassay showed an AUC of 0.89 to differentiate neuropathologically defined AD from non-AD in blood samples taken during life in one cohort [47], the corresponding number for plasma P-tau181 measured using Single molecule array (Simoa™) was 0.97 in another [49]. These findings call for further studies comparing different P-tau biomarkers in the same cohort. Interestingly, high plasma P-tau181 is found in tau PET-negative (Braak stage 0) individuals who have evidence of brain amyloidosis by amyloid PET [46], predicts subsequent AD dementia in cognitively unimpaired individuals and MCI patients [45], and plasma P-tau181 levels show a significant increase in pre-symptomatic familial AD mutation carriers 16 years before estimated symptom onset [50]. Collectively, these data suggest that plasma P-tau181 detects Aβ-induced tau pathophysiology years before tau pathology is detectable by PET.

## Fluid biomarkers for neurodegeneration

Whilst CSF T-tau might better reflect Aβ-induced tau secretion in AD rather than general neurodegeneration [39], neurofilament light (NfL) has emerged as a strong biomarker candidate for the latter [51]. The biomarker can be measured in both CSF and plasma (or serum), and the correlation between CSF and blood concentrations is good to excellent (r values of 0.70 to 0.97) [52]. The highest NfL levels are seen in frontotemporal, as well as vascular and HIV-associated dementias [53]. However, the findings in familial AD are also quite clear; mutation carriers show a sudden change in their blood NfL levels 10–15 years before expected clinical onset, which probably marks the onset and intensity of the neurodegenerative process [54, 55]. In sporadic AD, there is a clear association of increased plasma NfL concentration with Aβ and tau PET positivity, as well as with longitudinal neurodegeneration as determined by magnetic resonance imaging (MRI), but with a larger overlap across groups than in familial AD [56]. This might be due to the multitude of neurodegenerative changes that may cause NfL increase in people older than 70 years of age.

## From research tools to clinical implementation

In Europe, CSF biomarkers as supportive tools in the diagnostic evaluation of patients with suspected AD have been used in memory clinics since the early 2000s, but they have not yet been formally approved or recommended by regulatory authorities. Both the European Medical Agency (EMA) and the United States Food and Drug Administration (FDA) have encouraged the further study of CSF biomarkers in the context of clinical AD diagnostics, and the Coalition Against Major Diseases (CAMD) CSF Biomarker Team is working toward seeking formal qualification from the FDA on the use of CSF biomarkers for clinical trial enrichment at the pre-dementia stage of the disease [57]. Additionally, the Alzheimer’s Association has published Appropriate Use Criteria, i.e., specific clinical indications when the CSF tests are warranted in the diagnostic assessment of patients with suspected AD [58]. Standard operating procedures for pre-analytical sample handling have been agreed upon and published for both CSF [59] and plasma [22]. Reference methods and materials for CSF Aβ42 assay standardization [10], as well as high-precision clinical chemistry tests on fully automated instruments, are in place [60], which bodes well for full implementation of these biomarkers in clinical laboratory practice with uniform reference limits around the globe; in many European countries CSF biomarkers are already used in clinical laboratory practice in accordance with country-specific regulations. Work on the reference measurement procedures for CSF Aβ40, T-tau and P-tau181 is ongoing under the auspices of the IFCC CSF Proteins working group; the Aβ40 part of this work should be concluded during 2020. Similar work is now also being initiated for the blood tests. Plasma NfL is already an available test in clinical laboratory practice in Sweden, the Netherlands and France, and many clinical laboratories are now working towards validating plasma P-tau tests for clinical use.

## Limitations of fluid‐based biomarkers

A drawback of fluid biomarkers is the inability to determine brain region-specific changes, which may limit staging of disease severity and their use as progression markers. For plasma P-tau181, a step-wise increase with disease severity has been reported [46], and similar data have been reported for plasma NfL (with the caveat that this biomarker is not specific to AD progression) [56], but this is less clear for the other biomarkers; the CSF Aβ42/Aβ40 ratio, for example, appears to be a bimodal marker (normal or abnormal) without a clear relationship between the degree of change and the extent of the pathology [14]. Tau and Aβ PET imaging may be done to provide a more direct assessment of disease stage in select clinical cases and in clinical trials.

## Conclusions

We may see a regulatory approval of an Aβ-targeting drug in the near future. This class of treatments will likely be expensive and there will be a need to ensure that patients who are considered for the treatment actually do have the drug target. Synthesizing the recent biomarker breakthroughs above, it is relatively easy to envision blood-based testing for AD pathology using plasma Aβ42/Aβ40 ratio and plasma P-tau as screening tools. Positive patients could then be referred to a specialized memory clinic to be more closely examined, undergo amyloid PET imaging where available, and commence treatment with an anti-Aβ antibody therapy, if Aβ positivity is verified. Plasma P-tau (representing a neuronal reaction to Aβ) and NfL levels (representing neurodegeneration) could be monitored throughout the treatment (e.g., every third month, given the dynamics of NfL change after acute brain injury [61]), followed by yearly amyloid PET scans (given the observed amyloid PET changes in anti-Aβ clinical trials [62]). For anti-Aβ antibodies, repeat MRIs would be needed, at least initially, to monitor amyloid-related imaging abnormalities (ARIA) [63], but in the future, it is possible that increases in plasma NfL concentration could substitute for MRI to detect clinically relevant ARIA (this potential use needs to be formally examined, though). The patient could then be treated until amyloid PET is negative and plasma P-tau concentration has normalized. Post-treatment, the patient could be followed with annual plasma P-tau and NfL measurements to gauge the potential need for additional therapy. In our view, future clinical trials should incorporate both imaging and fluid biomarker approaches to assess biological response, at the same time as they provide the information needed to develop the most effective biomarker algorithm for treatment selection, dose optimization and drug monitoring.

## Data Availability

Not applicable.

## Abbreviations

AD: Alzheimer’s disease
Aβ: Amyloid β
T-tau: Total-tau
P-tau: Phosphorylated tau
NfL: Neurofilament light
CSF: Cerebrospinal fluid
APP: Amyloid precursor protein
PET: Positron emission tomography
ECL: Electrochemiluminescence
Simoa: Single molecule array
MRI: Magnetic resonance imaging
CAMD: Coalition Against Major Diseases
EMA: European Medical Agency
IFCC: International Federation of Clinical Chemistry and Laboratory Medicine
ARIA: Amyloid-related imaging abnormalities
